# Case Report: Eschar-like subcutaneous lesion in disseminated melioidosis revealing anti-interferon-γ autoantibody-associated adult-onset immunodeficiency and a review of the literature

**DOI:** 10.3389/fmed.2026.1955643

**Published:** 2026-09-09

**Authors:** Samadhi Patamatamkul

**Affiliations:** Department of Medicine, Suddhavej Hospital, Faculty of Medicine, Mahasarakham University, Maha Sarakham, Thailand

**Keywords:** anti-interferon-γ autoantibodies, *Burkholderia pseudomallei*, climate change, eschar, flooding

## Abstract

**Background:**

Melioidosis, caused by the environmental saprophyte *Burkholderia pseudomallei*, is acquired from soil and surface water and rises sharply in incidence after heavy rainfall and flooding—something that extreme-weather trends under climate change are expected to make more frequent and more widespread. The disease presents with a broad clinical spectrum, but eschars or ecthyma gangrenosum-like necrotic skin lesions are very rarely reported and may be mistaken for scrub typhus in coendemic regions.

**Case presentation:**

A 66-year-old woman from northeast Thailand with no known comorbidity presented with fever and chest pain 1 week after walking barefoot through floodwater. Contrast-enhanced computed tomography demonstrated mediastinal necrotic lymphadenitis and hepatosplenic microabscesses. Despite broad-spectrum β-lactam therapy, she remained febrile and, on illness day 8, developed a painful subcutaneous nodule that evolved within 2 days into a dark, eschar-like lesion. Pus culture grew *B. pseudomallei* that was resistant to ceftazidime and susceptible to imipenem and trimethoprim/sulfamethoxazole. Her fever and skin lesion resolved with meropenem. Because the disease was unusually aggressive and accompanied by recurrent, self-limiting panniculitis-like episodes, she was evaluated for adult-onset immunodeficiency and anti-interferon-γ autoantibodies were detected. She completed 1 month of meropenem followed by trimethoprim/sulfamethoxazole eradication therapy, and imaging at 3 months showed complete resolution.

**Conclusion:**

A structured review identified only six previously published patients with an eschar or eschar-like necrotic lesion in culture-confirmed melioidosis; the majority, as in our patient, had disseminated disease, and almost all had a documented soil or freshwater exposure. Melioidosis should be considered in patients with rapidly progressive visceral abscesses and necrotic skin lesions in the weeks after flooding or other heavy freshwater exposure in endemic regions, and unexplained severity should prompt evaluation for anti-interferon-γ autoantibody-associated immunodeficiency together with confirmation of unusual resistance phenotypes. As flooding becomes more frequent, clinical awareness and simple protective measures during and after floods are likely to be increasingly important.

## 1 Introduction

Melioidosis is a potentially life-threatening infection caused by the environmental Gram-negative bacillus *Burkholderia pseudomallei*, which is endemic across Southeast Asia and northern Australia. Infection is acquired through percutaneous inoculation, inhalation, or ingestion following exposure to contaminated soil and surface water, and incidence rises sharply after heavy rainfall and flooding (1, 2). The intensity of rainfall in the 2 weeks preceding admission independently predicts pneumonia, septic shock, and death, probably reflecting increased inhalation of aerosolized organisms during severe weather (3). Heavy rain and localized flooding also mobilize the organism from deeper soil into surface water and increase its output from water seeps; thus, increased case numbers have followed storms, cyclones, and consecutive very wet years (4). Because climate change is increasing the frequency and intensity of extreme rainfall across the tropics and is projected to expand the environmental range of the organism, melioidosis is expected to become both more common in endemic areas and more widely distributed (4, 5); modeling already indicates substantial under-recognition, with an estimated 165,000 cases and 89,000 deaths worldwide each year (5). The clinical spectrum ranges from localized skin infection to pneumonia, bacteremia, and disseminated visceral abscesses, particularly of the liver and spleen (1).

Cutaneous melioidosis most often manifests as localized ulcers, pustules, or subcutaneous abscesses. Eschars or ecthyma gangrenosum-like necrotic lesions are distinctly uncommon and, when reported, have generally accompanied severe disseminated infection (6–11). Because an eschar is classically regarded as a hallmark of rickettsial disease such as scrub typhus, this presentation is a genuine diagnostic challenge in regions where both pathogens circulate.

Anti-interferon-γ (IFN-γ) autoantibodies cause an acquired adult-onset immunodeficiency (AOID) that predisposes previously healthy adults, predominantly of Southeast Asian ancestry, to disseminated infection with intracellular pathogens including *B. pseudomallei* and non-tuberculous mycobacteria (12). Separately, resistance to ceftazidime—a first-line agent for the intensive phase of melioidosis therapy—is rare in *B. pseudomallei* but is clinically consequential (13, 14).

What is unique about the present case is the convergence of the following three individually rare features in a single patient: an eschar-like subcutaneous lesion, a ceftazidime-resistant isolate, and previously unrecognized anti-IFN-γ autoantibody-associated AOID unmasked by the severity of the episode. We describe the case and place it in the context of a structured review of published reports of eschars in culture-confirmed melioidosis.

## 2 Case description

### 2.1 Patient information and presentation

A 66-year-old Thai woman from northeast Thailand, a well-recognized melioidosis-endemic region, presented with fever, chills, and pleuritic chest pain of 1 day's duration. She had walked barefoot through floodwater 1 week earlier following heavy rainfall. She had no known comorbidity; no history of diabetes mellitus, corticosteroid use, or immunosuppressive therapy; and no family history of unusual or recurrent infection. Her recent medical history was notable only for right dacryocystitis with lacrimal duct stenosis, treated with a 7-day course of dicloxacillin. Approximately 2 months before admission, she had experienced painful subcutaneous nodules that resolved spontaneously without treatment; these had not been investigated.

On admission, her temperature was 38.6°C with tachycardia and mild hepatomegaly. There were no skin lesions, no lymphadenopathy, and no murmur or focal neurological deficit; the remainder of the examination was unremarkable.

### 2.2 Timeline

The chronology of the episode of care is summarized in Table 1.

**Table 1:** Timeline of the episode of care.

Time point	Clinical events and findings	Investigations	Management
2 months before admission	Painful subcutaneous nodules, self-limiting; not investigated	—	None
∼3 weeks before admission	Right dacryocystitis with lacrimal duct stenosis	—	Dicloxacillin, 7 days
7 days before admission	Walked barefoot through floodwater after heavy rainfall; asymptomatic	—	—
Day 1 (admission)	Fever of 38.6°C, chills, and pleuritic chest pain; tachycardia and mild hepatomegaly; no skin lesions	WBC 23,000/µL (84% neutrophils); AST/ALT/ALP 77/86/577 U/L; HbA1c 6.5%; blood cultures taken	Intravenous ceftriaxone 2 g q24h
Days 3–5	Persistent fever; no clinical improvement	Blood cultures: no growth (bacterial, fungal, or mycobacterial)	Escalated to piperacillin/tazobactam 4.5 g q6h
Days 5–7	Ongoing fever	Contrast-enhanced CT of chest/abdomen: rim-enhancing mediastinal and perihilar nodes with central low attenuation; hepatic and splenic microabscesses; abdominal lymphadenopathy (Figure 1)	Continued piperacillin/tazobactam
Day 8	Painful subcutaneous nodule appears	Initial IHA for *B. pseudomallei* 1:20	Continued piperacillin/tazobactam
Day 10	Nodule evolves into a dark, eschar-like lesion (Figure 2)	Incision and drainage: ∼3 mL pus; Gram stain negative	Changed to intravenous meropenem 1 g q8h
Day 13	Diagnosis established	Pus culture: *B. pseudomallei*—ceftazidime-resistant, imipenem and TMP/SMX susceptible (MicroScan MIC panel); ceftazidime resistance confirmed by disk diffusion, EUCAST v14.0	Meropenem continued
Day 20	Defervescence after 10 days of meropenem; skin lesion regressing	—	Meropenem continued
Day 25	Transient painful subcutaneous nodule, left thigh; resolved spontaneously	Anti-IFN-γ autoantibody-positive (1.13; OD cutoff <1); convalescent IHA >1:1,024	Meropenem continued
Week 4/discharge	Afebrile, lesion re-epithelialized without debridement	—	Completed 1 month of meropenem; started oral TMP/SMX (320/1,600 mg twice daily) eradication therapy
3 months	Asymptomatic, no relapse	Repeat CT: resolution of mediastinal lymphadenopathy and hepatosplenic lesions	TMP/SMX continued; long-term surveillance for opportunistic infection

ALP, alkaline phosphatase; ALT, alanine aminotransferase; AST, aspartate aminotransferase; CT, computed tomography; EUCAST, European Committee on Antimicrobial Susceptibility Testing; HbA1c, glycated hemoglobin; IFN-γ, interferon-γ; IHA, indirect hemagglutination assay; MIC, minimum inhibitory concentration; OD, optical density; TMP/SMX, trimethoprim/sulfamethoxazole; WBC, white blood cell count.

### 2.3 Diagnostic assessment

Initial laboratory testing showed leukocytosis (white blood cell count of 23,000/µL, with a neutrophil percentage of 84%) and a mild cholestatic-predominant elevation of liver enzymes (aspartate aminotransferase, alanine aminotransferase, and alkaline phosphatase levels of 77, 86, and 577 U/L, respectively) without hyperbilirubinemia. Glycated hemoglobin was 6.5%, consistent with newly recognized impaired glucose regulation. Blood, fungal, and mycobacterial cultures obtained before and during therapy ultimately showed no growth. Contrast-enhanced computed tomography (CT) of the chest and abdomen demonstrated multiple rim-enhancing mediastinal and perihilar lymph nodes with central low attenuation, numerous microabscesses in the liver and spleen, and abdominal lymphadenopathy—findings consistent with necrotic lymphadenitis and disseminated abscess formation (Figure 1).

**Figure 1:**
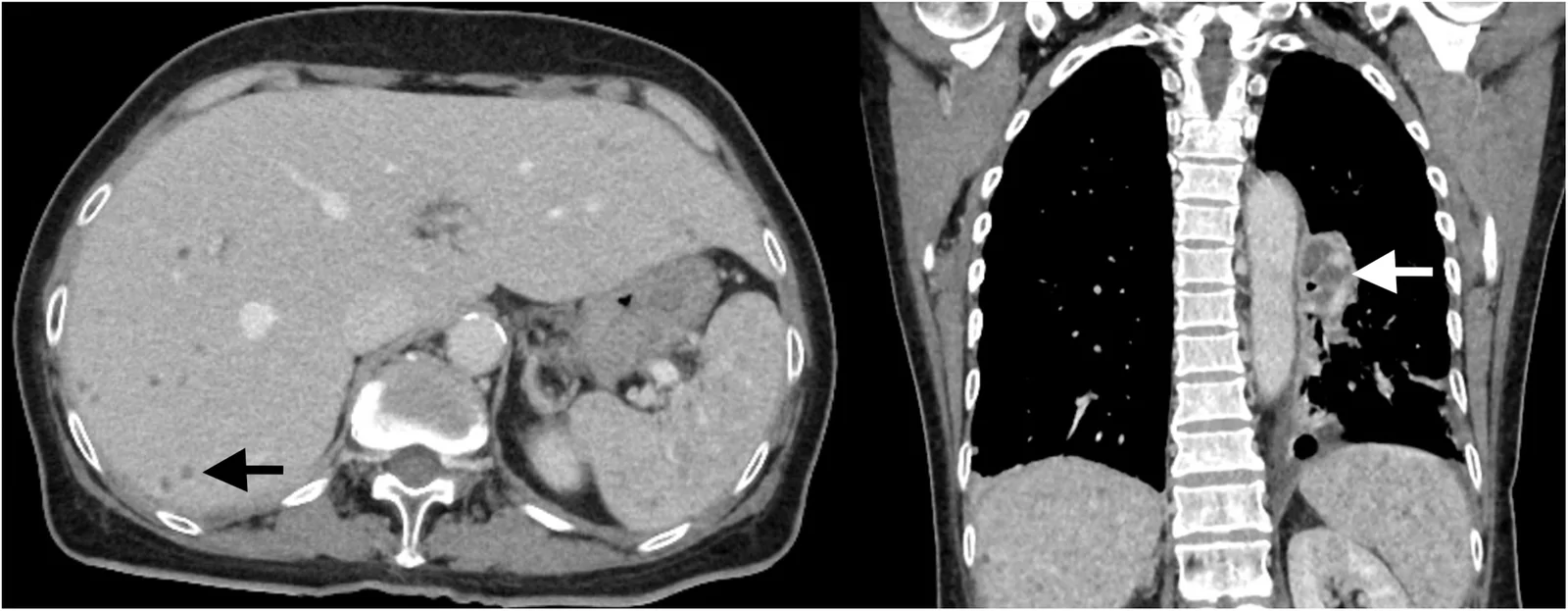
Contrast-enhanced computed tomography shows multiple enlarged necrotic mediastinal lymph nodes with peripheral rim enhancement in the left perihilar, subcarinal, subaortic, para-aortic, and right lower paratracheal regions, measuring up to 1.5 cm in diameter and suggestive of necrotic lymphadenopathy with abscess formation (white arrowhead). Diffuse hepatosplenomegaly is also present, with innumerable ill-defined hypodense lesions of varying sizes scattered throughout the hepatic and splenic parenchyma, some demonstrating peripheral enhancement, consistent with multiple microabscesses (black arrowhead).

The differential diagnosis at this stage included disseminated tuberculosis, disseminated non-tuberculous mycobacterial infection, disseminated endemic yeasts (histoplasmosis and/or talaromycosis), pyogenic liver and splenic abscesses from hematogenous spreading of purulent bacteria (*Staphylococcus aureus* and/or *Salmonella* spp.), and disseminated melioidosis. The diagnostic challenges were substantial: repeated blood cultures were sterile, the initial indirect hemagglutination assay (IHA) titer for *B. pseudomallei* on illness day 8 was low (1:20), and a Gram stain of drained pus showed no organisms. The diagnosis was ultimately established microbiologically. Culture of pus aspirated from the eschar-like lesion grew *B. pseudomallei*, reported on a Gram-negative MicroScan minimum inhibitory concentration (MIC) panel (Beckman Coulter, Inc., West Sacramento, CA, USA) as resistant to ceftazidime (MIC ≥ 32 µg/mL) and susceptible to imipenem (MIC ≤ 1 µg/mL) and trimethoprim/sulfamethoxazole (TMP/SMX) (MIC ≤ 2/38 µg/mL); ceftazidime resistance was independently confirmed by disk diffusion (zone diameter, 16 mm; resistance breakpoint, <18 mm) interpreted using European Committee on Antimicrobial Susceptibility Testing (EUCAST) v14.0 breakpoints (15). A convalescent IHA titer rose to >1:1,024. Given the unusually aggressive disseminated course in a host without classical risk factors, together with the recurrent self-limiting panniculitis-like episodes, testing for anti-IFN-γ autoantibodies was performed and returned positive (reported value 1.13; optical density cutoff <1), establishing a diagnosis of AOID.

### 2.4 Therapeutic intervention

Empirical intravenous ceftriaxone 2 g every 24 h was started on admission and escalated on day 3 to piperacillin/tazobactam 4.5 g every 6 h without clinical improvement. On illness day 8, the patient developed a painful subcutaneous nodule that evolved over 2 days into a dark, eschar-like lesion (Figure 2). Incision and drainage beneath the lesion yielded approximately 3 mL of pus. On the basis of the imaging pattern, the floodwater exposure history, and the progressive necrotic skin findings, therapy was changed on day 10 to intravenous meropenem 1 g every 8 h to target melioidosis—a change made before culture confirmation and retained once the ceftazidime-resistant isolate was reported. Meropenem was continued for a total intensive phase of 1 month, followed by oral TMP/SMX (trimethoprim 320 mg/sulfamethoxazole 1,600 mg twice daily) as eradication therapy, planned for a minimum of 3 months. No corticosteroid or immunomodulatory therapy was given.

**Figure 2:**
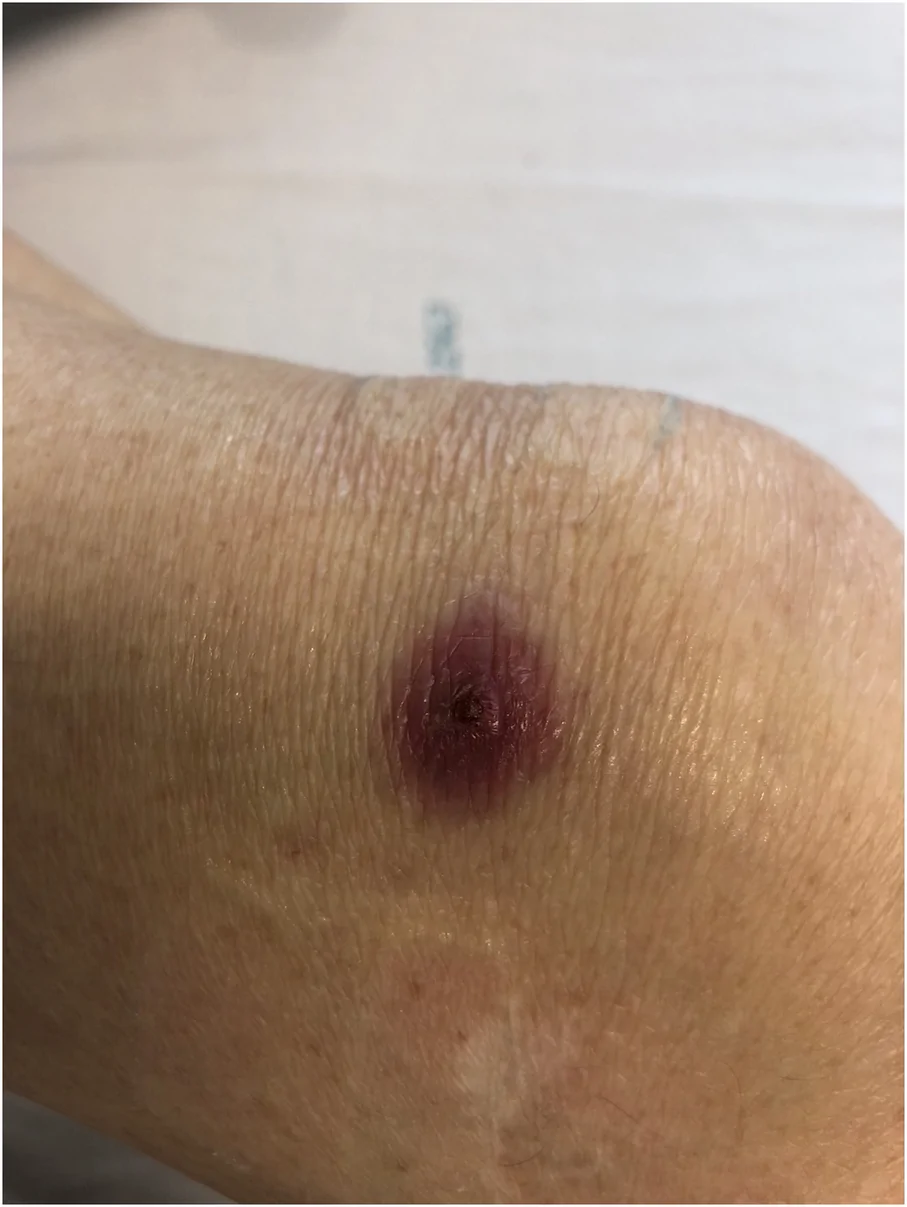
A painful, solitary, well-demarcated, erythematous-to-violaceous oval patch with a central necrotic papule overlying a subcutaneous abscess, resembling an eschar, is shown on the superolateral aspect of the right knee.

### 2.5 Follow-up and outcomes

The patient’s fever resolved after 10 days of meropenem and the eschar-like lesion demarcated and desloughed progressively, re-epithelializing without the need for further surgical debridement or skin grafting. On hospital day 25, she developed a transient painful subcutaneous nodule on the left thigh that resolved spontaneously, resembling her preadmission episodes. She was discharged in good condition and tolerated TMP/SMX without hematological or renal adverse effects. Repeat CT at 3 months showed resolution of the mediastinal lymphadenopathy and hepatosplenic lesions, and she remained asymptomatic and relapse-free with normal inflammatory markers. She continues under long-term surveillance for opportunistic infection given the diagnosis of AOID.

## 3 Discussion

This case describes disseminated melioidosis with an eschar-like subcutaneous lesion following floodwater exposure, in which the unusual severity of the illness led to the identification of anti-IFN-γ autoantibody-associated AOID and discovery of a ceftazidime-resistant isolate.

A structured search of PubMed and Scopus performed on 17 July 2026, using (*melioidosis* OR *Burkholderia pseudomallei*) AND (eschar* OR “eschar-like” OR “ecthyma gangrenosum”), supplemented by a broader database search using cutaneous- and skin-related terms, retrieved 265 records (PubMed 91; Scopus 174). After removal of 73 duplicates, 192 records were screened in Rayyan; 185 were excluded and seven were included. Among the seven records initially included, two were a photo-quiz question and its corresponding answer describing the same patient; these two records were merged and counted as one case; a further record—a 1996 report of melioidosis presenting as fever of unknown origin with multiple pustular or necrotic skin and subcutaneous lesions—was excluded at this stage because the full text could not be retrieved and no patient-level data were extractable from the abstract alone. Five eligible reports therefore remained from the database searches. A supplementary manual search using the same keywords, together with reference list and citation examinations, identified one further report not indexed in either database, resulting in six published patients (6–11). Only reports describing an eschar, eschar-like, or ecthyma gangrenosum-like necrotic lesion in culture-confirmed disease were included; non-specific cutaneous melioidosis and lesions attributable to a coinfection of scrub typhus were excluded. The full eligibility criteria, exact database queries, screening rules, and selection flow are provided in the Supplementary Material. Including the present case, the clinical courses of seven patients are summarized in Table 2.

**Table 2:** Reported patients with an eschar or eschar-like necrotic lesion in culture-confirmed melioidosis, in chronological order.

Case/reference	Age/sex	Country, exposure, and host factors	Presentation and extent of disease	Eschar or necrotic lesion	Microbiology	Treatment	Outcome
Nussbaum et al. (6)	21 y/M	Georgia, USA; hurled into a clay embankment in a drainage area of a sewage treatment plant during a road traffic accident; sewage plant worker; no travel to an endemic area	Multiple facial fractures with soil contamination of the left orbit; corneal ulceration and perforation requiring enucleation; no systemic dissemination	Necrosis and sloughing of the left upper eyelid (localized, post-traumatic)	Conjunctival swab from the anophthalmic orbit 8 weeks after injury: *Pseudomonas pseudomallei* (former name of *B. pseudomallei*), biochemically confirmed by the CDC	Fracture reduction, conjunctival flaps and enucleation; parenteral and topical antibiotics directed at concurrent pathogens	Survived; eye lost
Apisarnthanarak et al. (7)	48 y/F	Thailand; diabetes mellitus	Fever with widespread skin lesions; bacteremia; abdominal ultrasonography showed no intra-abdominal abscess	Multiple ecthyma-like lesions distributed over the neck, face, trunk, arms and legs	Skin lesion and two blood cultures: *B. pseudomallei*, susceptible to ceftazidime, cefoperazone/sulbactam, TMP/SMX, doxycycline and chloramphenicol; antibody titer 1:320	Intravenous ceftazidime + TMP/SMX 14 days → doxycycline, chloramphenicol and TMP/SMX for 20 weeks	Full recovery
Boruah et al. (8)	16 months/F	Northeast India; near-drowning in drain water 7 days before illness	Septicemic pneumonia with bilateral consolidation, cavitation and abscesses; multiple erythematous nodules and ulcers	Multiple (“a few eschars”), ecthyma gangrenosum-like	Blood culture: *B. pseudomallei*	Piperacillin/tazobactam + netilmicin → meropenem	Died
Saonanon et al. (9)	48 y/M	Northern Thailand; farmer; previous pulmonary tuberculosis and chronic obstructive pulmonary disease	Right orbital cellulitis progressing to necrotizing fasciitis; bacteremia with multiple small liver and splenic abscesses; elevated liver enzymes	Single erythematous-edged necrotic forehead lesion with multiple small abscesses	Blood and lesion pus: *B. pseudomallei*, susceptible to ceftazidime, TMP/SMX, and imipenem	Intravenous ceftazidime 14 days with daily debridement → oral TMP/SMX + doxycycline for 6 months	Full recovery; vision preserved, residual forehead scar
Ramanathan et al. (10)	49 y/F	Tamil Nadu, India; farmer; newly diagnosed diabetes (HbA1c 9.2%)	Fever, cough, and breathlessness; disseminated abscesses in the lungs, liver, spleen, and brain	Single 3 cm × 2 cm ulceronecrotic forehead eschar	Blood, pus, and tissue: *B. pseudomallei*	Meropenem + supportive care	Died
Palaniappan et al. (11)	5 months/F	South Andaman, Andaman, and Nicobar Islands, India; fisherman community	Fever, severe respiratory distress, bilateral purulent ear discharge, and diarrhea; anemia, thrombocytopenia, deranged liver and renal function; lesion initially mimicking scrub typhus	Single necrotic eschar-like lesion on the right thigh	Blood culture: *B. pseudomallei*, susceptible to meropenem, TMP/SMX, ceftazidime, and chloramphenicol; scrub typhus IgM negative	Intravenous meropenem 21 days → oral TMP/SMX to complete a 6-week eradication phase	Full recovery
Present case	66 y/F	Northeast Thailand; barefoot floodwater exposure after heavy rain; anti-IFN-γ autoantibody-associated AOID	Mediastinal and perihilar necrotic lymphadenitis with hepatosplenic microabscesses; blood cultures sterile	Single eschar-like subcutaneous lesion on the superolateral aspect of the right knee	Pus culture: *B. pseudomallei*, resistant to ceftazidime and susceptible to imipenem and TMP/SMX; paired IHA 1:20 → >1:1,024	Meropenem 1 g q8 h for 1 month → oral TMP/SMX eradication therapy	Full recovery

AOID, adult-onset immunodeficiency; CDC, Centers for Disease Control and Prevention; F, female; HbA1c, glycated hemoglobin; IFN-γ, interferon-γ; IHA, indirect hemagglutination assay; M, male; TMP/SMX, trimethoprim/sulfamethoxazole; y, years.

Cases are listed chronologically. The 2005 report was published as a photo quiz and a separate answer describing the same patient; these were merged and counted once.

The seven patients ranged in age from a 5-month-old infant and a 16-month-old child to adults aged 21–66 years and were reported from Thailand, India, and the United States. Exposures included near-drowning in drain water, agricultural soil and water contact, floodwater exposure, and traumatic inoculation of clay soil in a road traffic accident; recognized host risk factors—diabetes mellitus, chronic lung disease, and, in the present case, anti-IFN-γ autoantibodies—were present in several. Six of the seven patients had disseminated or bacteremic disease with pulmonary, visceral, or multifocal involvement. One did not: a post-traumatic orbital case in which the organism was recovered from an anophthalmic orbit 8 weeks after injury, without systemic infection, and in which causal attribution of the eyelid necrosis to the organism is uncertain (6). An eschar therefore accompanies disseminated disease in the majority of reported patients but is not invariably a marker of it, and localized postinoculation necrosis is also described. Solitary lesions were reported in five patients and multiple lesions in two. These involved the forehead, eyelid, thigh, and knee or were widely distributed over the trunk and limbs. Outcomes diverged sharply: the two patients with fulminant bacteremic pulmonary disease died despite escalation to meropenem (8, 10), whereas the other five patients all survived. In each survivor, the necrotic lesion either prompted an early switch to targeted therapy or provided the specimen that yielded the organism, which suggests that the prognostic weight of an eschar lies less in the lesion itself than in how quickly it is recognized as a symptom of melioidosis.

Environmental exposure was the proximate cause in this patient and in almost every reported case, and it is the point at which the disease is most readily prevented. Six of the seven patients had a documented exposure—floodwater, drain water, agricultural soil and water, or soil driven into a wound during trauma—and in the present case, symptoms began 1 week after the patient waded barefoot through floodwater, an interval consistent with percutaneous inoculation. Floods concentrate this risk; they mobilize *B. pseudomallei* from deeper soil into surface water and carry it into homes and streets, exposing large numbers of people, often barefoot and often with minor skin breaks, over a short period (4). As extreme rainfall events become more frequent and more intense, both the incidence of melioidosis in endemic regions and its emergence in areas previously regarded as non-endemic are expected to rise (2, 4, 5). Two practical implications follow. Clinicians working in flood-affected areas should include melioidosis in the differential diagnosis of febrile illness, sepsis, and necrotic skin lesions in the weeks following a flood. Equally, preflood public health messaging should promote footwear and waterproof covering of wounds, particularly for people with diabetes, chronic kidney or liver disease, or a known immune defect—a group that, as this case demonstrates, may not know they belong to it.

The pathogenesis of eschar formation in melioidosis remains uncertain because histopathological data are scarce. A plausible mechanism is small-vessel thrombosis or localized intravascular coagulation triggered by bacterial invasion in a predisposed host, including hosts with unrecognized immune defects such as AOID, as in this case. A plausible pathophysiological sequence in this case is that neutralizing anti-IFN-γ autoantibodies disrupt IFN-γ-induced STAT1 phosphorylation, thereby impairing classical macrophage activation and intracellular antimicrobial activity (12). Because IFN-γ-activated macrophages restrict intracellular *B. pseudomallei* through reactive oxygen- and nitrogen-dependent mechanisms, this impairment may permit bacterial persistence and replication and facilitate subsequent dissemination (16, 17). The resulting increase in local bacterial burden may provoke a secondary, partly IFN-γ-independent innate inflammatory response, including TNF-α and IL-1β release, endothelial activation, platelet adhesion, and local microvascular coagulation (18). Microvascular thrombosis and consequent tissue ischemia could therefore contribute to central necrosis and eschar formation (18). However, intracellular hyper-replication and microvascular thrombosis were not directly demonstrated in this patient; this pathway should therefore be regarded as biologically plausible rather than established. Increased IFN-γ and TNF-α production has also been observed in stimulated T cells from patients with anti-IFN-γ autoantibodies and may represent compensatory immune dysregulation, although its contribution to localized thrombosis remains uncertain (19). Clinically, an eschar should not be treated as specific for rickettsial infection in a patient from a melioidosis-endemic region, particularly when pneumonia, septicemia, multifocal abscesses, diabetes, or environmental water or soil exposure is present. Cultures of both blood and the lesion remain essential for etiological attribution, and in this case, the lesion—not the blood—yielded the organism.

With respect to susceptibility testing, ceftazidime-resistant *B. pseudomallei* remains uncommon, reported in approximately 0.15% of isolates in a large series from northeast Thailand, with resistance mostly attributed to a membrane-bound, chromosomally located Ambler class A β-lactamase *penA* mutation (13); resistance has remained rare over two decades of surveillance in the same region (14). However, the following three other mechanisms are recognized: elevated *penA* expression due to a promoter mutation (−78G > A) (20, 21); a gene duplication and amplification (GDA) event in the *penA* chromosomal region (22, 23); and *penA* point mutations, including C69Y (20, 22, 24, 25), P167S (24, 26, 27), and D240G (21). P167S is the best-supported determinant; this omega-loop substitution enhances ceftazidime hydrolysis, and replacing the mutant allele with wild-type *penA* in a resistant clinical isolate reduced the ceftazidime MIC 64-fold and restored susceptibility (13, 27). A GDA event increases *penA* expression and β-lactamase activity but appears to be a weaker determinant, as expression and enzyme activity correlate imprecisely with the ceftazidime MIC and amplification alone does not invariably confer resistance (13). Resistance is not confined to *penA*; deletion of the β-lactam target penicillin-binding protein 3 (PBP3) has been described in ceftazidime-resistant Thai isolates (28), and elevated expression of the class D β-lactamase gene *oxa* has been observed in laboratory-generated ceftazidime-induced mutants (29). The ceftazidime-resistant, carbapenem-susceptible phenotype in our patient is compatible with a *penA*-mediated mechanism, as PenA variants such as P167S principally expand ceftazidime hydrolysis while imipenem activity is generally preserved (13, 26); meropenem was not included in our automated panel, and published reports conflict as to whether these variants also reduce meropenem susceptibility (13, 24). Because the isolate was not retained, neither whole-genome sequencing nor targeted *penA* sequencing (coding sequence plus promoter region) could be performed, and the mechanism underlying resistance in this case remains undetermined. Where ceftazidime resistance is encountered, we would now recommend preserving the isolate for *penA* sequencing, for which rapid real-time PCR assays are available (26), as identifying a *penA* determinant would support an early switch to a carbapenem.

Recognizing this phenotype is clinically consequential and underscores the value of confirming unexpected results, as was done here by disk diffusion interpreted against EUCAST breakpoints. Prior β-lactam exposure has been associated with the emergence of resistance, which in surveillance data was detected a median of 15 days after the start of therapy (14). Our patient had recently received dicloxacillin; however, that agent has no established role in selecting ceftazidime-hydrolyzing PenA variants, and resistant isolates have also been recovered long after β-lactam therapy was completed (13, 22). Any causal contribution therefore remains speculative. Interpretation of TMP/SMX susceptibility likewise warrants care, particularly by disk diffusion, because zone-edge reading is difficult and false resistance has been described. Published data directly comparing automated MIC systems with reference broth microdilution for β-lactam testing of *B. pseudomallei* are limited; in resource-limited settings, confirming unexpected or discordant results by a second method is a pragmatic safeguard.

Finally, the detection of anti-IFN-γ autoantibodies provided a unifying explanation for the severity and atypical cutaneous manifestations of this infection, and it carries important implications for long-term surveillance (12). AOID should be considered in adults from endemic regions who present with unexpectedly severe or disseminated melioidosis, recurrent infection, or infection with multiple intracellular pathogens (30).

### 3.1 Strengths and limitations

The strengths of this report are culture confirmation of the pathogen from the necrotic lesion, independent confirmation of the ceftazidime-resistant phenotype by a second method, serological confirmation by paired IHA titers, documented radiological resolution at 3 months, and a transparent, reproducible literature search with prespecified eligibility criteria. The limitations are those inherent to a single case: histopathology of the eschar was not obtained, so the vascular mechanism proposed above remains inferential; whole-genome sequencing was not performed, so *penA*-mediated resistance could not be confirmed and the contribution of prior dicloxacillin exposure remains speculative; broth microdilution, the reference method, was unavailable; and anti-IFN-γ autoantibody titers were not measured serially, so their trajectory during recovery is unknown. The literature review was limited by the small number of eligible reports, by markedly incomplete patient-level reporting in the oldest of them (6), by the exclusion of one otherwise relevant 1996 report whose full text could not be retrieved, and by a single-reviewer design without a second independent screener.

### 3.2 Key takeaways

First, in a febrile patient from a melioidosis-endemic region, a necrotic eschar—particularly following floodwater or soil exposure and accompanied by evidence of visceral involvement—should raise suspicion for melioidosis alongside scrub typhus. Frontline clinicians should not treat presumptively for scrub typhus alone. While awaiting microbiological results, empirical therapy active against both pathogens should be initiated when both diagnoses remain plausible, using doxycycline plus an intravenous anti-melioidosis agent, i.e., meropenem for critically ill patients or ceftazidime for those without critical illness. The eschar should also be sampled for culture even when blood cultures are sterile. Second, in the reported literature, an eschar has invariably signaled disseminated rather than localized disease and should trigger imaging of the chest and abdomen. Third, unexpectedly severe or recurrent melioidosis in an adult without classical risk factors warrants testing for anti-IFN-γ autoantibodies. Fourth, an unexpected ceftazidime-resistant result should be confirmed by a second method rather than dismissed because it changes the first-line therapy. Fifth, melioidosis is a climate-sensitive infection; flood exposure should be taken as a specific epidemiological cue at the bedside, and counseling about soil and freshwater contact before each rainy season is part of the long-term care of any patient found to have an underlying immune defect.

## Patient perspective

The patient reflected that she had been very frightened by how difficult her illness was to diagnose, and that she had never imagined walking through floodwater could cause such a severe disease. Learning that she has an underlying immune defect has made her far more conscious of environmental exposure, and she intends to protect herself from soil and water contact in order to prevent further infections. She was very glad that the disease finally resolved, and she said she will certainly attend her follow-up appointments to make sure that no new episodes of infection develop.

## Data Availability

The original contributions presented in the study are included in the article/Supplementary Material, further inquiries can be directed to the corresponding author.
