# Supplementary material for: Case Report: Eschar-like subcutaneous lesion in disseminated melioidosis revealing anti-interferon-γ autoantibody-associated adult-onset immunodeficiency and a review of the literature
Source: Front Med (Lausanne). 2026 Sep 9;13:1955643. doi: 10.3389/fmed.2026.1955643 (PMC13597349; doi:10.3389/fmed.2026.1955643)
Supplement: Supplementary file 1 [file Table1.docx]

**CARE Checklist of information to include when writing a case report**

**Manuscript:** Case Report: Eschar-like subcutaneous lesions in disseminated melioidosis revealing anti–interferon-γ autoantibody–associated adult-onset immunodeficiency and a review of the literature

**Author:** Samadhi Patamatamkul **Journal:** Frontiers in Medicine (Infectious Diseases: Pathogenesis and Therapy)

**Checklist version:** CARE Checklist (2013). ✔ = item reported. Locations refer to numbered sections of the submitted manuscript.

| **Topic** | **Item** | **Checklist item description** | **Reported — section / location** |
| --- | --- | --- | --- |
| **Title** | **1** | The diagnosis or intervention of primary focus followed by the words "case report" | ✔ Title: "Case Report: Eschar-like subcutaneous lesions in disseminated melioidosis revealing anti–interferon-γ autoantibody–associated adult-onset immunodeficiency and a review of the literature" (title page) |
| **Key Words** | **2** | 2 to 5 key words that identify diagnoses or interventions in this case report, including "case report" | ✔ Keywords (title page): *Burkholderia pseudomallei*; eschar; ecthyma gangrenosum; anti–interferon-γ autoantibodies; adult-onset immunodeficiency |
| **Abstract**  **(no references)** | **3a** | Introduction: What is unique about this case and what does it add to the scientific literature? | ✔ Abstract, "Background" paragraph |
|  | **3b** | Main symptoms and/or important clinical findings | ✔ Abstract, "Case presentation", sentences 1–3 |
|  | **3c** | The main diagnoses, therapeutic interventions, and outcomes | ✔ Abstract, "Case presentation", sentences 4–6 |
|  | **3d** | Conclusion — What is the main "take-away" lesson(s) from this case? | ✔ Abstract, "Conclusion" paragraph |
| **Introduction** | **4** | One or two paragraphs summarizing why this case is unique (may include references) | ✔ Section 1 (Introduction), paragraphs 1–4; uniqueness stated explicitly in paragraph 4; references (1–8) |
| **Patient Information** | **5a** | De-identified patient specific information | ✔ Section 2.1, paragraph 1 (age, sex, region only; no identifiers, no dates of care) |
|  | **5b** | Primary concerns and symptoms of the patient | ✔ Section 2.1, paragraph 1 (fever, chills, pleuritic chest pain of one day's duration) |
|  | **5c** | Medical, family, and psycho-social history including relevant genetic information | ✔ Section 2.1, paragraph 1 (no comorbidity, no diabetes, no corticosteroid or immunosuppressive use, no relevant family history of unusual infection; barefoot floodwater exposure) |
|  | **5d** | Relevant past interventions with outcomes | ✔ Section 2.1, paragraph 1 (dicloxacillin 7 days for right dacryocystitis; prior self-limiting subcutaneous nodules ~2 months earlier); Table 1, rows 1–2 |
| **Clinical Findings** | **6** | Describe significant physical examination (PE) and important clinical findings | ✔ Section 2.1, paragraph 2 (T 38.6 °C, tachycardia, mild hepatomegaly, no skin lesions at presentation); evolution of lesions in Section 2.4; Figure 2 |
| **Timeline** | **7** | Historical and current information from this episode of care organized as a timeline | ✔ Section 2.2 and Table 1 (Timeline of the episode of care), from 2 months before admission to 3-month follow-up |
| **Diagnostic Assessment** | **8a** | Diagnostic testing (such as PE, laboratory testing, imaging, surveys) | ✔ Section 2.3, paragraphs 1–2 (haematology, liver enzymes, HbA1c, blood/fungal/mycobacterial cultures, contrast-enhanced CT, IHA, pus Gram stain and culture, MicroScan MIC panel, EUCAST disk diffusion, anti–IFN-γ autoantibody assay); Figure 1; Table 1 |
|  | **8b** | Diagnostic challenges (such as access to testing, financial, or cultural) | ✔ Section 2.3, paragraph 2 (sterile blood cultures, low initial IHA titre 1:20, negative Gram stain); Section 3.1 (reference broth microdilution and whole-genome sequencing unavailable in a resource-limited setting) |
|  | **8c** | Diagnosis (including other diagnoses considered) | ✔ Section 2.3, paragraph 2 (differential: disseminated tuberculosis, lymphoma, disseminated nontuberculous mycobacterial infection, pyogenic liver abscess, melioidosis); final diagnosis: disseminated melioidosis with ceftazidime-resistant B. pseudomallei and anti–IFN-γ autoantibody–associated AOID |
|  | **8d** | Prognosis (such as staging in oncology) where applicable | ✔ Section 2.5 and Section 3 (disseminated disease; literature outcomes contrasted in Table 2); Section 2.5 (ongoing relapse and opportunistic-infection risk due to AOID) |
| **Therapeutic Intervention** | **9a** | Types of therapeutic intervention (such as pharmacologic, surgical, preventive, self-care) | ✔ Section 2.4 (pharmacological: ceftriaxone, piperacillin–tazobactam, meropenem, TMP/SMX; procedural: incision and drainage of the eschar-like lesion; preventive: long-term eradication therapy and surveillance) |
|  | **9b** | Administration of therapeutic intervention (such as dosage, strength, duration) | ✔ Section 2.4 (ceftriaxone 2 g q24h; piperacillin–tazobactam 4.5 g q6h; meropenem 1 g q8h for 1 month; TMP/SMX 320/1,600 mg twice daily for ≥3 months); Table 1 |
|  | **9c** | Changes in therapeutic intervention (with rationale) | ✔ Section 2.4 (escalation on day 3 for non-response; switch to meropenem on day 10 based on imaging pattern, exposure history and necrotic skin lesions, retained after ceftazidime-resistant isolate reported); Table 1 |
| **Follow-up and Outcomes** | **10a** | Clinician and patient-assessed outcomes (if available) | ✔ Clinician-assessed: Section 2.5 (defervescence, lesion re-epithelialization, radiological resolution at 3 months). Patient-assessed: Section 4 (Patient perspective) |
|  | **10b** | Important follow-up diagnostic and other test results | ✔ Section 2.3 (convalescent IHA >1:1024) and Section 2.5 (repeat CT at 3 months showing resolution of mediastinal and hepatosplenic lesions; normal inflammatory markers); Table 1, final row |
|  | **10c** | Intervention adherence and tolerability (How was this assessed?) | ✔ Section 2.5 (full course completed; TMP/SMX tolerated, assessed by clinical review and by monitoring of complete blood count and renal function at scheduled outpatient visits) |
|  | **10d** | Adverse and unanticipated events | ✔ Section 2.5 (no haematological or renal adverse effects of TMP/SMX; unanticipated transient left-thigh subcutaneous nodule on hospital day 25, resolved spontaneously); Table 1 |
| **Discussion** | **11a** | A scientific discussion of the strengths AND limitations associated with this case report | ✔ Section 3.1 (Strengths and limitations) |
|  | **11b** | Discussion of the relevant medical literature with references | ✔ Section 3, paragraphs 2–6; Table 2 (seven patients compared, including six published reports); references (1–8); full search methodology, screening rules and selection flow in the Supplementary Material |
|  | **11c** | The scientific rationale for any conclusions (including assessment of possible causes) | ✔ Section 3, paragraphs 4–6 (proposed small-vessel thrombosis mechanism; penA-mediated ceftazidime resistance and possible role of prior β-lactam exposure; anti–IFN-γ autoantibodies as a unifying explanation for severity), with uncertainty acknowledged in Section 3.1 |
|  | **11d** | The primary "take-away" lessons of this case report (without references) in a one paragraph conclusion | ✔ Section 3.2 (Take-away lessons) |
| **Patient Perspective** | **12** | The patient should share their perspective in one to two paragraphs on the treatment(s) they received | ✔ Section 4 (Patient perspective) |
| **Informed Consent** | **13** | Did the patient give informed consent? Please provide if requested | ✔ Yes ☐ No — Ethics statement: written informed consent obtained from the patient for participation and for publication of this case report and accompanying clinical images. Signed consent form available to the editorial office on request. |

All 13 CARE items (including all sub-items) are reported. The completed checklist is submitted as a supplementary file in accordance with Frontiers Case Report requirements. Further information on the CARE guidelines is available at https://www.care-statement.org.
