# Supplementary material for: Case Report: Eschar-like subcutaneous lesion in disseminated melioidosis revealing anti-interferon-γ autoantibody-associated adult-onset immunodeficiency and a review of the literature
Source: Front Med (Lausanne). 2026 Sep 9;13:1955643. doi: 10.3389/fmed.2026.1955643 (PMC13597349; doi:10.3389/fmed.2026.1955643)
Supplement: Supplementary file 2 [file Table2.docx]

**Supplementary Material**

**Case Report: Eschar-like subcutaneous lesion in disseminated melioidosis revealing anti–interferon-γ autoantibody–associated adult-onset immunodeficiency and a review of the literature**

Samadhi Patamatamkul

# **Supplementary Methods: structured literature review**

This Supplementary Material reports, in full, the objective, eligibility criteria, exact reproducible database queries, screening process, and results of the structured literature review summarized in section 3 and Table 2 of the main article.

## **1 Objective and eligibility criteria**

**Objective.** To identify human reports in which an eschar, eschar-like lesion, or ecthyma-gangrenosum–like necrotic lesion was clinically described in culture-confirmed melioidosis caused by *Burkholderia pseudomallei*.

**Included.** Case reports, case series, and cohorts with extractable patient-level eschar data; all ages; any geographic setting; any publication language; no date restriction.

**Excluded.** Reviews without new cases; animal or laboratory studies; reports explicitly stating that no eschar was present; nonspecific cutaneous melioidosis without an eschar or necrotic-eschar description; coinfections in which the eschar was attributable to scrub typhus rather than to *B. pseudomallei*; and reports for which no full text could be retrieved and from which no patient-level data could be extracted.

**Note on nomenclature.** Reports published under the former name *Pseudomonas pseudomallei* were eligible where the biochemical profile reported by the authors matched *B. pseudomallei*; one such report (Nussbaum et al., 1980) was included on this basis.

## **2 Search strategy**

Exact reproducible searches were run on 17 July 2026 and are shown in Supplementary Table S1. No date or language restriction was applied in the principal searches.

**Supplementary Table S1.** Exact database queries used on 17 July 2026.

| **Database** | **Exact query** | **Purpose / note** |
| --- | --- | --- |
| PubMed | (("Melioidosis"[Mesh] OR melioidosis[Title/Abstract] OR "*Burkholderia pseudomallei*"[Title/Abstract]) AND (eschar*[All Fields] OR "eschar-like"[All Fields] OR "ecthyma gangrenosum"[All Fields])) | Principal high-specificity search. |
| PubMed (supplementary) | (("Melioidosis"[Mesh] OR melioidosis[Title/Abstract] OR "*Burkholderia pseudomallei*"[Title/Abstract]) AND (cutaneous[Title/Abstract] OR skin[Title/Abstract]) AND ("case reports"[Publication Type] OR "case report"[Title/Abstract] OR "case series"[Title/Abstract] OR cohort[Title/Abstract])) | Broader screen to capture reports in which "eschar" appeared only in the full text. |
| Scopus | TITLE-ABS-KEY ( melioidosis OR "*Burkholderia pseudomallei*" ) AND TITLE-ABS-KEY ( eschar* OR "eschar-like" OR "ecthyma gangrenosum" ) | Principal title/abstract/keyword search. |
| Scopus (supplementary) | TITLE-ABS-KEY ( melioidosis OR "*Burkholderia pseudomallei*" ) AND TITLE-ABS-KEY ( cutaneous OR skin ) AND TITLE-ABS-KEY ( "case report" OR "case series" OR cohort ) | Broader screen plus reference and citation checking. |

**Records retrieved:** PubMed 91; Scopus 174; total 265. Exports from both databases were imported into Rayyan (https://www.rayyan.ai) for de-duplication and screening.

**Supplementary manual search.** Because case reports of melioidosis are frequently published in regional journals that are not indexed in either database, an additional manual internet search (Google and Google Scholar) was performed using the same keyword combinations (melioidosis OR *Burkholderia pseudomallei*; eschar, eschar-like, ecthyma gangrenosum, necrotic skin lesion). This identified one further eligible report (Palaniappan et al., 2025) that was present in neither PubMed nor Scopus. Reference lists of all included articles and of narrative reviews of cutaneous melioidosis were hand-searched, and forward citation checking was performed for each included article.

## **3 Study selection and data extraction**

Records retrieved by the four queries were combined in Rayyan and de-duplicated: of 265 imported records, 73 were removed as duplicates and 2 flagged pairs were resolved as non-duplicates, leaving 192 unique records for screening.

**Two-step screening rule.** (i) Where a title and abstract were available and contained no mention of a cutaneous, skin, or subcutaneous lesion, the record was excluded at the title/abstract stage. (ii) Where any cutaneous, skin, or subcutaneous involvement was mentioned anywhere in the title or abstract, the full text was retrieved and read in order to determine whether the lesion described was compatible with an eschar or a necrotic eschar-like lesion, or was instead a non-necrotic ulcer, pustule, abscess, or cellulitis. Only lesions of the former type qualified for inclusion. Records without a retrievable abstract were carried forward to full-text assessment by default.

Applying this rule, 185 of the 192 records were excluded and 7 were included. Two of the seven included records were the photo-quiz question and the corresponding published answer describing the same patient (Apisarnthanarak et al., 2005); these were merged and counted as a single case. A further included record (Handa et al., Br J Clin Pract 1996;50:116–7), which reported melioidosis presenting as fever of unknown origin with multiple pustular or necrotic skin and subcutaneous lesions, was subsequently excluded because the full text could not be retrieved and no age, sex, lesion morphology, treatment, or outcome data were extractable from the abstract alone. Five eligible reports therefore remained from the database searches. Adding the one report identified by the supplementary manual search gives six published patients; with the present case, seven patients were available for synthesis.

Screening, full-text assessment, and data extraction were performed by the single author. Because the review was performed by one investigator, no inter-rater agreement statistic is reported, and this is acknowledged as a limitation in section 3.1 of the main article.

For each included report the following variables were extracted into a structured form: first author and year; patient age and sex; country and exposure or epidemiological context; comorbidity or immune status; clinical presentation and extent of dissemination; eschar characteristics (single or multiple, site, size, morphology); specimen(s) yielding *B. pseudomallei* and reported antimicrobial susceptibility; antimicrobial and adjunctive treatment; and outcome. Where a variable was not stated in the source article it was recorded as "not reported" (NR) rather than inferred.

## **4 Results of the review**

Six published human reports met all eligibility criteria (references 2–7 of the main article), spanning 1980 to 2025 and originating from Thailand, India, and the United States. Records were excluded at the full-text stage principally because the cutaneous lesion was a non-necrotic ulcer, pustule, abscess, or cellulitis without an eschar or necrotic-eschar description; because melioidosis was not culture-confirmed; because the article was a review without new patient-level data; or because an eschar was present but attributable to concurrent scrub typhus. Adding the present case, seven patients were available for synthesis; their characteristics are tabulated in Table 2 of the main article. One of the seven had localized post-traumatic disease without systemic dissemination, and this is discussed in section 3 of the main article. Patient-level data were least complete for the oldest report (Nussbaum et al., 1980). The selection flow is summarized in Supplementary Table S2.

**Supplementary Table S2.** Study selection flow.

| **Stage** | **Records** | **Detail** |
| --- | --- | --- |
| Records identified — PubMed | 91 | Principal and supplementary PubMed queries, run 17 July 2026. |
| Records identified — Scopus | 174 | Principal and supplementary Scopus queries, run 17 July 2026. |
| Total imported into Rayyan | 265 | Combined exports from both databases. |
| Duplicates removed | 73 | 71 duplicate pairs resolved and removed; 2 flagged pairs confirmed as non-duplicates and retained. |
| Unique records screened | 192 | Title/abstract screening against the two-step rule described in section 3. |
| Excluded at title/abstract or full text | 185 | No cutaneous, skin, or subcutaneous lesion mentioned; or lesion described but not an eschar or necrotic eschar-like lesion; or melioidosis not culture-confirmed; or review without new patient-level data; or eschar attributable to concurrent scrub typhus. |
| Included at screening | 7 | Records passing both screening steps. |
| Merged as one patient | −1 | The photo-quiz question and its published answer (Apisarnthanarak et al., 2005) describe the same patient and were counted once. |
| Excluded — full text unobtainable | −1 | Handa et al., 1996: no full text retrievable and no patient-level data extractable from the abstract. |
| Eligible from database searches | 5 | Nussbaum 1980; Apisarnthanarak 2005; Boruah 2013; Saonanon 2013; Ramanathan 2025. |
| Added by supplementary manual search | 1 | Palaniappan et al., 2025 — indexed in neither PubMed nor Scopus; identified by manual internet search and confirmed by full-text review. |
| Published reports included | 6 | Nussbaum 1980; Apisarnthanarak 2005; Boruah 2013; Saonanon 2013; Ramanathan 2025; Palaniappan 2025. |
| Total patients synthesized | 7 | 6 published patients + the present case (Table 2 of the main article). |

## **5 Definitions used**

**Eschar.** A localized area of dark, adherent necrotic tissue, with or without a surrounding erythematous halo.

**Ecthyma-gangrenosum–like lesion.** A lesion evolving from an erythematous macule or nodule to a haemorrhagic bulla or pustule and then to a necrotic ulcer with a central black slough.

**Culture-confirmed melioidosis.** Isolation of *B. pseudomallei* from any clinical specimen (blood, pus, tissue, respiratory, or other sterile site).

**Disseminated disease.** Involvement of two or more non-contiguous organ systems, or bacteraemia with any distant focus.

# **Supplementary Data files**

**Supplementary File 1.** Completed CARE (CAse REport) checklist for this manuscript, submitted as a separate file.
